# Phase analysis single-photon emission computed tomography (SPECT) myocardial perfusion imaging (MPI) detects dyssynchrony in myocardial scar and increases specificity of MPI

**DOI:** 10.1186/s13550-019-0476-y

**Published:** 2019-01-31

**Authors:** John P. Bois, Chris Scott, Panithaya Chareonthaitawee, Raymond J. Gibbons, Martin Rodriguez-Porcel

**Affiliations:** 1 Department of Cardiovascular Medicine, Mayo Clinic, Rochester, MN USA; 2 Department of Biostatistics, Mayo Clinic, Rochester, MN USA; 3 Department of Cardiovascular Diseases, Mayo Clinic College of Medicine, 200 First Street SW, Rochester, MN 55905 USA

**Keywords:** Coronary artery disease, Nuclear cardiology and PET, Diagnostic testing

## Abstract

**Background:**

Myocardial perfusion imaging (MPI) with single-photon emission computed tomography (SPECT) is commonly used to assess patients with cardiovascular disease. However, in certain scenarios, it may have limited specificity in the identification of hemodynamically significant coronary artery disease (e.g., false positive), potentially resulting in additional unnecessary testing and treatment. Phase analysis (PA) is an emerging, highly reproducible quantitative technology that can differentiate normal myocardial activation (synchrony) from myocardial scar (dyssynchrony). The objective of this study is to determine if PA can improve the specificity SPECT MPI.

**Methods:**

An initial cohort of 340 patients (derivation cohort), referred for SPECT-MPI, was prospectively enrolled. Resting MPI studies were assessed for resting perfusion defects (scar). These were utilized as the reference standard for scar. Subsequently, we collected a second independent validation cohort of 138 patients and tested the potential of PA to reclassify patients for the diagnosis of “scar” or “no scar.” Patients were assigned to three categories depending upon their pre-test probability of scar based on multiple clinical and imaging parameters: ≤ 10% (no scar), 11–74% (indeterminate), and ≥ 75% (scar). The ability of PA variables to reclassify patients with scar to a higher group and those without scar to a lower group was then determined using the net reclassification index (NRI).

**Results:**

Entropy (≥ 59%) was independently associated with scar in both patient cohorts with an odds ratio greater than five. Furthermore, when added to multiple clinical/imaging variables, the use of entropy significantly improved the area under the curve for assessment of scar (0.67 vs. 0.59, *p* = 0.04). The use of entropy correctly reclassified 24% of patients without scar, by clinical model, to a lower risk category (as determined by pre-test probability) with an overall NRI of 18% in this validation cohort.

**Discussion:**

The use of PA entropy can improve the specificity of SPECT MPI and may serve as a useful adjunctive tool to the interpreting physician. The current study determined the optimal PA parameters to detect scar (derivation cohort) and applied these parameters to a second, independent, patient group and noted that entropy (≥ 59%) was independently associated with scar in both patient cohorts. Therefore, PA, which requires no additional imaging time or radiation, enhances the diagnostic capabilities of SPECT MPI.

**Conclusion:**

The use of PA entropy significantly improved the specificity of SPECT MPI and could influence the labeling of a patient as having or not having myocardial scar and thereby may influence not only diagnostic reporting but also potentially prognostic determination and therapeutic decision-making.

## Background

Coronary artery disease (CAD) remains the leading cause of mortality in the USA [1]. To accurately diagnose and risk stratify patients with CAD, it is imperative that clinicians have access to diagnostic imaging techniques that are not only sensitive but also specific.

Nine million myocardial perfusion imaging (MPI) examinations are performed annually in the USA [2] with a sensitivity and specificity for the detection of CAD of approximately 85% and 70%, respectively [3]. Results of MPI testing have critical diagnostic, prognostic, and therapeutic ramifications [4–9].

Despite the excellent sensitivity of MPI, its specificity has been called into question. Motion artifact [10], excessive subdiaphragmatic activity [11], breast attenuation [12], and asymmetric ventricular wall thickening [13] can lead to perceived perfusion defects and thereby decrease specificity [14]. This, in turn, may result in possibly unnecessary invasive procedures and medical treatment with potentially increased morbidity and mortality. Furthermore, given the approximate $2000 cost per MPI study [15], some authors have concluded that the “risk-benefit ratio for stress testing is not convincing.” [3] These challenges call for further refinement of MPI, to optimize sensitivity and specificity of these studies.

Phase analysis (PA) is an automated [16], highly reproducible [17], and repeatable [18] software application which can be applied to traditional gated SPECT MPI. It utilizes the partial volume effect [19] and Fourier first harmonics to assess the onset of myocardial contraction at over 600 myocardial locations thereby determining myocardial synchrony [16]. It is independent of the type of imaging camera [20], reconstruction algorithm [21], or tracer dose utilized [22]. PA has been shown to detect dyssynchrony in the heart failure population [23] and may be of value in suspected CAD. Specifically, myocardial infarction will result in regional left ventricular (LV) contractile disparity and hence myocardial dyssynchrony [24–26], even before there is visual regional myocardial contractility dysfunction.

The objectives of the current study are the following: First, to determine the presence of dyssynchrony, by PA, in patients with myocardial scar on resting SPECT MPI. Second, to define optimal PA dyssynchrony parameters, from a derivation cohort, which will have the greatest specificity for the detection of scar. Third, to apply these criteria to a separate cohort (validation cohort) to determine the clinical utility (degree of patient reclassification, scar vs. no scar) that results when PA is added to the original SPECT MPI results as determined by expert analysis.

## Methods

### Patient selection

The study was approved by the Mayo Clinic Institutional Review Board (IRB). Patients with known or suspected CAD who were referred for SPECT MPI at a single tertiary referral center (Mayo Rochester) between August 2014 and November 2016 were eligible for the study. Those patients who underwent SPECT MPI between August 2014 and September 2015 were assigned to the derivation cohort; patients enrolled between October 2015 and November 2016 were assigned to the validation cohort. Patients were included if they were (1) ≥ 18 years of age, (2) were clinically referred for SPECT MPI, and (3) consented to study participation. Patients with atrial fibrillation/flutter, left bundle branch block (LBBB), paced rhythm, chronic resynchronization therapy (CRT), or depressed left ventricular ejection fraction (LVEF) were intentionally included in this study to permit broad application of the study’s findings. Demographic, clinical, and imaging data was abstracted from the clinical records on all patients.

### SPECT image acquisition protocol and image processing

Resting blood pressure, heart rate, and electrocardiogram (ECG) were obtained for all patients. For resting images, 8–10 mCi technetium-99m (^99m^Tc) sestamibi was intravenously administered. Forty-five to 60 minutes following injection, patients underwent upright (seated) and semi-supine gated imaging. All data was acquired utilizing a D-SPECT camera (Spectrum Dynamics, Haifa, Israel). Images were obtained as each of the nine pixelated detector columns rotated along its vertical axis and scanned the region of myocardium which was designated by the user. Sixteen frames per cardiac cycle were obtained, and data from each detector was stored in a 16 × 64 matrix with data from the nine detectors combined for final image reconstruction. Emission data was obtained via nine low energy, tungsten square hole collimators. Images were acquired using a standard 20% energy window centered on the 140 keV photopeak of ^99m^Tc.

Studies were processed using Spectrum Dynamics proprietary reconstruction algorithms (Quantitative Perfusion SPECT-QPS-, Cedars-Sinai Medical Center, Los Angeles) on a dedicated Spectrum Dynamics workstation. Reconstruction algorithm utilized the maximum-likelihood expectation-maximization (MELM) method with resolution recovery, 4–7 iterations and 32 subsets. An additional kernel convolution smoothing filter (Gaussian) was used on transaxial data. No attenuation or scatter correction was applied.

### Systolic function and perfusion interpretation

Quantitative Gated SPECT (QGS) software package was utilized to automatically calculate left ventricular (LV) end-diastolic volume, LV end-systolic volume, and LVEF from the short-axis images as previously described [27]. Images were displayed in three planes (short-axis, horizontal long-axis, and vertical long-axis) and subsequently divided into 17 segments [28]. QPS analysis was implemented to create LV myocardial perfusion maps that included automated calculation of the summed rest score (SRS). Scar quantification (SQ) was assessed by utilizing a previously validated method which entails calculating the fraction of a myocardial profile at five short-axis slices from the apex to the base of the heart and labeling the fraction of the profiles falling below a threshold value of 60% as scar [29].

In our laboratory, the determination to report a resting defect was predicated upon the integration of both clinical factors and imaging data by the study interpreter. Specifically, each study was reviewed in the three available planes (in both the supine and semi-supine position) by a consensus of an experienced nuclear cardiologist and nuclear cardiac radiologist (each with at least 10 years of experience) utilizing both qualitative and quantitative assessment of perfusion defects. Stress and rest perfusion data was simultaneously interpreted. QGS data was incorporated to assess for regional wall motion abnormalities, and QPS data was reviewed to note the automated SRS and IQ scores. In cases of disagreement between the automated perfusion data and the clinical interpretation, priority was given to the clinical interpretation. Following integration of this information, a final perfusion score for each segment was assigned by consensus. Defects that were felt to be artifact (attenuation or other) such as those involving the proximal septum or the inferior wall and those with completely normal wall thickening were reported as normal. A previously described 5-point scoring system was used to assess each of the 17 cardiac segments (4 = absent, 3 = severely diminished, 2 = moderately diminished, 1 = mildly diminished, and 0 = normal) [30]. This final SRS, which takes into account the quantitative SRS but is not necessarily the same as it can be modified by the interpreting physician, was calculated by adding the scores of all 17 segments. Any score > 0 was considered scar as this is reported as such to the referring physician at our institution. Furthermore, the objective of the current study is to assess the presence of dyssynchrony in any size perfusion defect. Clinical reporting of a resting perfusion defect was considered the “reference standard” for detection of scar. Mild scar was defined as a SRS of 1–4, moderate 5–8, and severe > 8. Both physicians evaluating myocardial perfusion were blinded to the PA data.

### Phase analysis

As described previously, a 3-D count distribution was obtained from the LV short-axis dataset and subjected to Fourier analysis resulting in the generation of a phase distribution completely encompassing the R-R interval (0–360°) [16]. Utilizing automated software (QGS 3.0; Cedars-Sinai Medical Center, Los Angeles CA), a phase histogram and polar map were created portraying the onset of myocardial contraction (OMC) for greater than 600 points in LV myocardium. Three indices of LV synchrony were automatically calculated from the phase histogram: phase histogram bandwidth (PHB) which portrays the range of degrees of the cardiac cycle during which myocardium is initiating contraction [31], phase standard deviation (PSD) which represents the standard deviation of the phase distribution [31], and entropy which is a measure of the variability in the histogram [32]. One of the study authors (JB) reviewed the PA data and was blinded to corresponding perfusion studies and interpretations.

### Pertinent clinical and imaging variables

Clinical risk factors which demonstrated correlation with obstructive CAD in the NCDR Cath-PCI Registry were assessed for each patient. These included age, gender, hyperlipidemia, insulin dependent diabetes mellitus, peripheral vascular disease, smoking history, family history of premature CAD, and presentation of typical angina symptoms [33]. Since the NCDR did not include other variables that were significant in the Duke databank [34], history of CAD (any degree), prior history of myocardial infarction (including ST and non-ST elevation myocardial infarction), and the presence of Q waves or ST depression on resting ECG were included as pertinent variables, along with QPS SRS. Regardless of their association with scar in the current small study population, all of the above variables were included in the clinical model [35].

### Statistical analysis

Categorical variables were summarized by count and percentage and were compared between groups using Pearson chi-square test or Fisher exact test, where appropriate. Distributions of continuous variables were examined for normality. Variables found to be approximately normally distributed were summarized by mean and standard deviation and compared between groups using two-sample *t* test. Continuous variables found to be non-normally distributed were summarized by median and quartiles and compared between groups using non-parametric rank-sum test. Univariable logistic regression analysis was initially performed to assess whether an association between dyssynchrony parameters (PHB, PSD and entropy) and the presence or absence of scar was present. Results were summarized as odds ratio (OR) and associated 95% confidence intervals. Receiver-operator characteristic (ROC) analysis was used to determine optimal thresholds, weighted for higher specificity in order to minimize false-positive results as has been described [36], for each of the dyssynchrony parameters for the detection of scar within the derivation cohorts. The dyssynchrony parameters at optimal thresholds were then evaluated in addition to the clinical and imaging parameters using logistic regression in both the derivation and the validation cohorts. We then assessed the improvement in ROC with dyssynchrony parameters compared to pertinent clinical and imaging risk factors alone. Lastly, reclassification of the patients’ diagnosis of scar or no scar by PA variables was assessed in the validation cohort. Specifically, patients were assigned to three categories based upon their pre-test probability of scar as calculated by the designated clinical and imaging parameters: ≤ 10% (low risk or no scar), 11–74% (indeterminate), and ≥ 75% (high risk or scar). The ability of PA variables to reclassify patients with scar to a higher group and those without a scar to a lower group was then determined by calculating the net reclassification index (NRI) [37]. Analyses were completed using SAS version 9.4 (SAS Institute Inc.). Statistical significance was set a priori at *p* < .05 and two-sided *p* values were used.

## Results

### Total study population—baseline variables

**Fig. 1:**
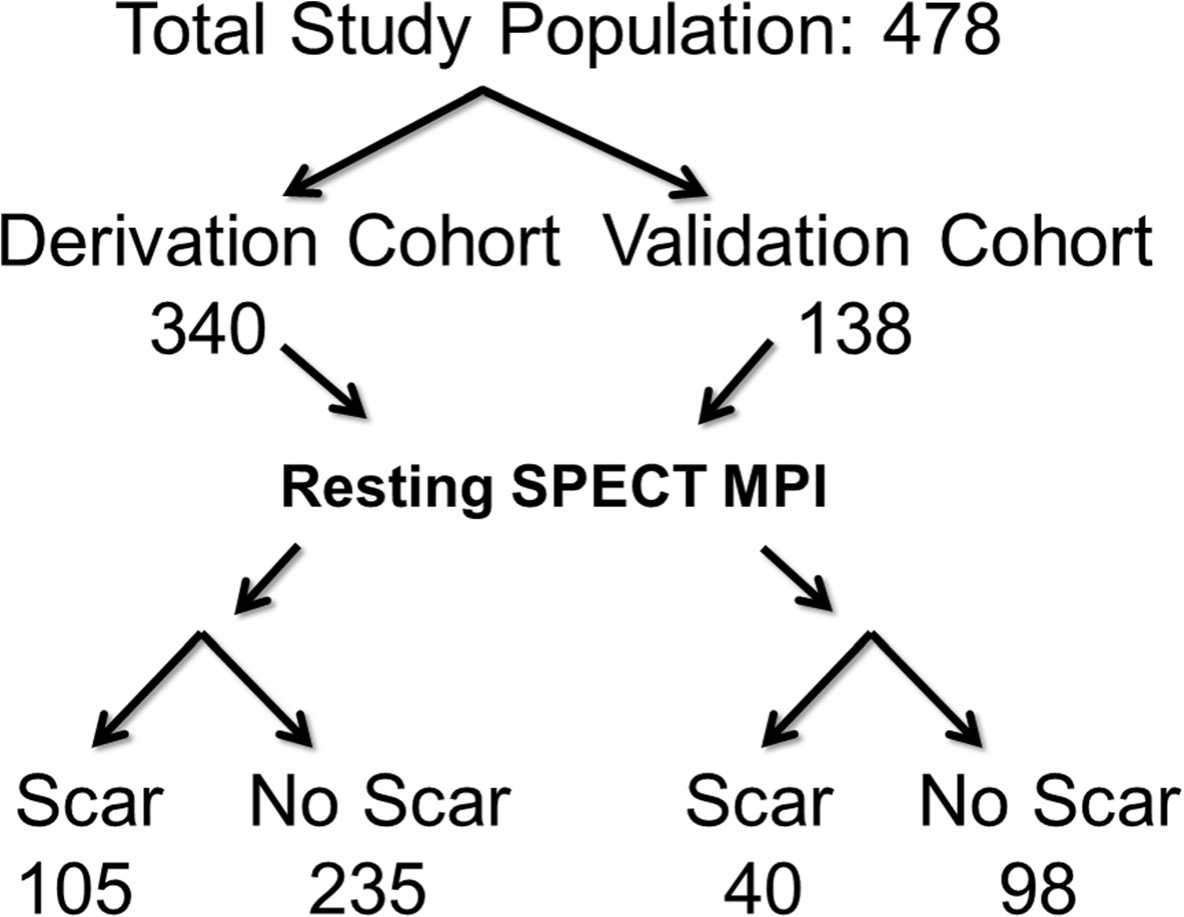
Study design. The total study population was 478 patients. Patients enrolled between August 2014 and September 2015 were included in the training group. Patients enrolled from October 2015 to November 2016 were included in the validation group (138 total). Both groups underwent resting SPECT MPI and were subsequently classified as having a scar or not having a scar based upon their resting SPECT MPI interpretation. Abbreviations: MPI, myocardial perfusion imaging; SPECT, single-photon emission computed tomography.

There was a total of 478 patients in the study, 340 in the initial cohort (derivation), and 138 in the second cohort (validation) (Fig. 1). Baseline clinical, laboratory, and imaging variables between the two cohorts were similar (Table 1). The majority of the patients were male with a mean age of 67–68 years. Approximately half of the patient population had a history of coronary artery disease, two-thirds hypertension and hyperlipidemia, and one quarter of the patients had diabetes mellitus (7% insulin dependent). Mean LVEF by SPECT (60%) was the same between both the derivation and the validation cohorts.

**Table 1:** Baseline clinical, laboratory, and imaging variables between the two cohorts

Variable	Derivation (*N* = 340)	Validation (*N* = 138)	*p* value
Scar, *n* (%)	105 (31%)	40 (29%)	0.68
Demographics			
Age	67.3 (12.3)	68.3 (12.0)	0.40
Male gender, *n* (%)	232 (68%)	98 (71%)	0.55
Caucasian, *n* (%)	310 (91%)	126 (91%)	0.96
Past medical history			
Coronary artery disease, *n* (%)	161 (47%)	64 (46%)	0.85
STEMI/NSTEMI, *n* (%)	50 (15%)	18 (13%)	0.64
Prior PCI, *n* (%)	96 (28%)	32 (23%)	0.26
Prior CABG, *n* (%)	42 (12%)	19 (14%)	0.67
HFrEF, *n* (%)	51 (15%)	23 (17%)	0.65
HFpEF, *n* (%)	7 (2%)	5 (4%)	0.32
ICD, *n* (%)	10 (3%)	1 (1%)	0.14
CRT, *n* (%)	1 (0%)	0 (0%)	0.52
Pacemaker, *n* (%)	12 (4%)	4 (3%)	0.73
Hypertension, *n* (%)	215 (63%)	86 (62%)	0.85
Hyperlipidemia, *n* (%)	236 (69%)	86 (62%)	0.13
Smoking, *n* (%)	167 (48%)	57 (42%)	0.33
OSA, *n* (%)	65 (19%)	34 (25%)	0.18
COPD, *n* (%)	21 (6%)	6 (4%)	0.43
Diabetes, insulin dependent, *n* (%)	27 (8%)	7 (5%)	0.27
Dialysis, *n* (%)	6 (2%)	1 (1%)	0.39
Peripheral vascular disease	41 (12%)	16 (12%)	0.89
Atrial fibrillation/atrial flutter, *n* (%)	57 (17%)	33 (24%)	0.07
Ventricular tachycardia, *n* (%)	6 (2%)	4 (3%)	0.43
Family history of premature CAD, *n* (%)	46 (14%)	14 (10%)	0.31
Valve disease (moderate or greater), *n* (%)	28 (14%)	10 (11%)	0.50
Medications			
Aspirin, *n* (%)	211 (62%)	96 (70%)	0.12
Beta-blocker, *n* (%)	187 (55%)	75 (54%)	0.90
Ticagreloror/plavix/prasugrel, *n* (%)	53 (16%)	21 (15%)	0.92
Statin, *n* (%)	222 (65%)	84 (61%)	0.36
CCB, *n* (%)	60 (18%)	28 (20%)	0.50
Diuretic, *n* (%)	105 (31%)	46 (33%)	0.60
ECG/imaging			
ECG Q wave, *n* (%)	17 (5%)	4 (3%)	0.31
ECG ST depression, *n* (%)	19 (6%)	6 (4%)	0.58
ECG paced, *n* (%)	19 (6%)	9 (7%)	0.69
ECG LBBB, *n* (%)	18 (5%)	13 (9%)	0.09
ECG atrial fibrillation/atrial flutter, *n* (%)	31 (9%)	20 (14%)	0.08
Nuclear EF	59.6 (11.9)	60.4 (12.4)	0.53
QPS SRS, median (Q1, Q3)	0.5 (0.0, 3.0)	0.0 (0.0, 2.0)	0.20
Clinical Presentation			
Typical symptoms, *n* (%)	28 (8%)	13 (10%)	0.62

Continuous variables expressed as mean ± standard deviation for symmetric data and median (interquartile range) for asymmetric data. Categorical variables expressed as count and percentage of patients

*Abbreviations*: *CABG* coronary artery bypass grafts, *CAD* coronary artery disease, *COPD* chronic obstructive pulmonary disease, *CRT* cardiac resynchronization therapy; *ECG* electrocardiogram, *ICD* implantable cardioverter defibrillator, *HFpEF* heart failure with preserved ejection fraction, *HFrEF* heart failure with reduced ejection fraction, *LBBB* left bundle branch block, *LVEF* left ventricular ejection fraction, *NSTEMI* non-ST elevation myocardial infarction, *OSA* obstructive sleep apnea, *PCI* percutaneous intervention, *QPS* Quantitative Perfusion SPECT, *SRS* summed rest score, *STEMI* ST elevation myocardial infarction

### Derivation cohort

One hundred and five patients (31%) in the derivation cohort had scar as detected by SPECT MPI. All three PA parameters, entropy, PHB, and PSD, were associated with scar on univariable analysis (Table 2). Specifically, all three PA parameters were progressively higher (indicating greater dyssynchrony) as SRS increased, indicating greater extent and severity of the perfusion defect (Table 3). ROC analysis was then utilized to determine the thresholds for each PA variable to optimize specificity for the detection of scar with the resulting variables ranging in specificity from 86% to 91% (Table 4).

**Table 2:** Derivation cohort—association between scar and phase analysis, univariable analysis

Variable	No scar (*N* = 235)	Scar (*N* = 105)	*p* value
Entropy (%)	45.0 (39.0, 51.0)	51.0 (45.0, 62.0)	< .001
Phase histogram bandwidth (°)	42.0 (36.0, 60.0)	54.0 (42.0, 96.0)	< .001
Phase standard deviation (°)	11.9 (8.5, 19.5)	17.4 (10.6, 25.0)	< .001

Continuous variables expressed as median (interquartile range)

**Table 3:** Derivation cohort—perfusion defect severity and dyssynchrony

Variable	Mild (1–4)^a^ (*N* = 66)	Moderate (5–8)^b^ (*N* = 18)	Severe (8+)^c^ (*N* = 21)	*p* value
Entropy (%)	48.5 (43.0, 56.0)	55.5 (47.0, 71.0)	62.0 (55.0, 69.0)	< .001
Phase histogram bandwidth (°)	48.0 (36.0, 72.0)	75.0 (42.0, 138.0)	96.0 (72.0, 138.0)	< .001
Phase standard deviation (°)	12.7 (9.9, 22.0)	19.7 (11.2, 37.6)	23.5 (17.8, 27.8)	0.002

Continuous variables expressed as median (interquartile range)

^a^Mild scar defined as summed rest score of 1–4

^b^Moderate scar defined as summed rest score of 5–8

^c^Severe scar defined as summed rest score > 8

**Table 4:** Derivation cohort—phase variables sensitivity and specificity

Variable	Sensitivity (*N*) (*N* = 235)	Specificity (*N* = 105)	AUC
Entropy ≥ 59%	35.2 (37/105)	90.6 (213/235)	0.629
Phase histogram bandwidth ≥ 78°	38.1 (40/105)	85.6 (202/235)	0.620
Phase standard deviation ≥ 26.7°	23.8 (25/105)	90.6 (213/235)	0.572

*Abbreviations*: *AUC* area under the curve

**Fig. 2:**
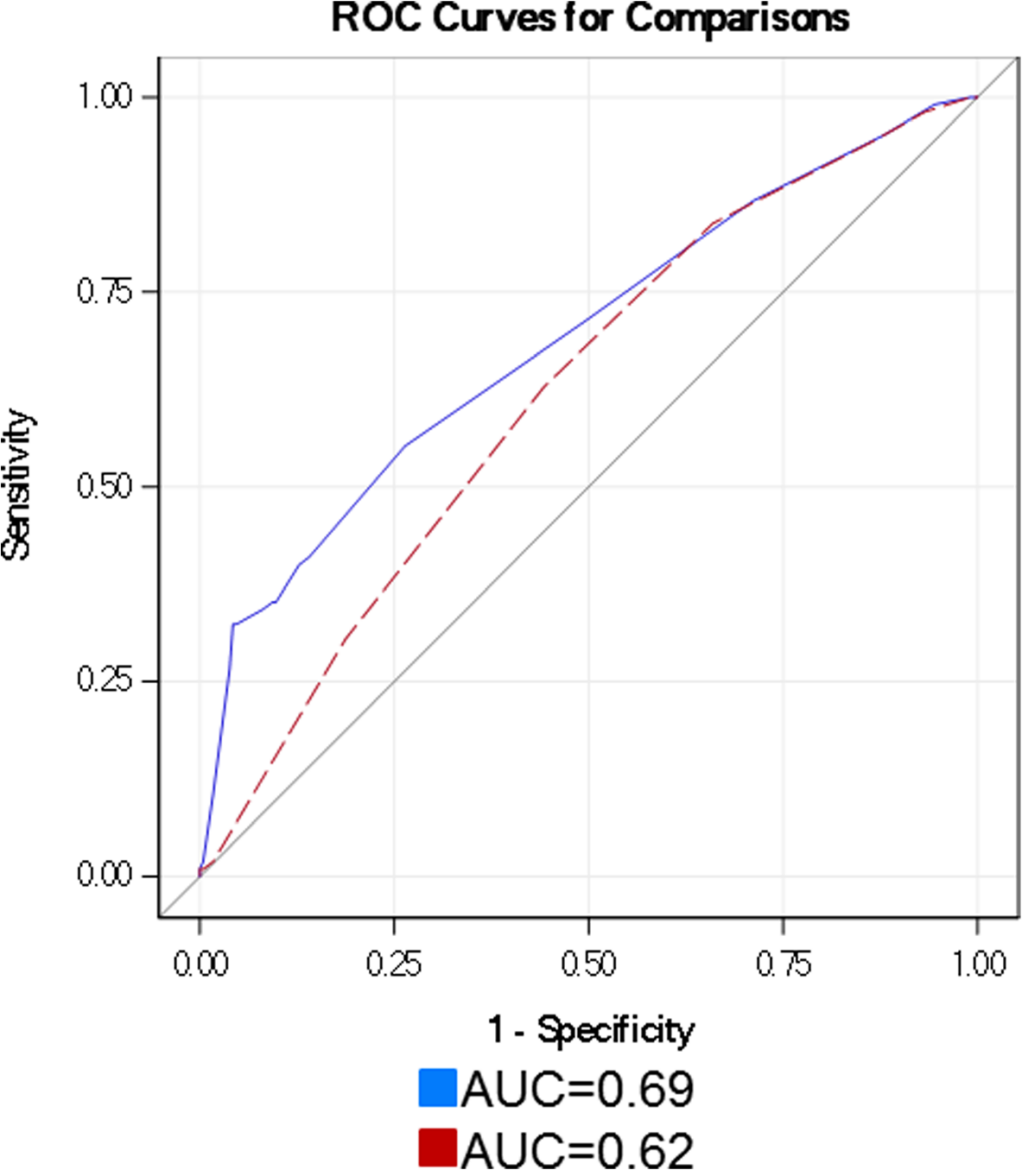
AUC difference estimate, 0.07 (0.02, 0.11), *p* = 0.005. *Abbreviations*: AUC area under the curve, ROC receiver operator curve

Each of the phase analysis parameters was independently associated with scar on multivariable analysis (Table 5). When compared to clinical/imaging variables, entropy had the greatest association with scar with the highest odds ratio (OR = 5.04). Clinical/imaging variable models were evaluated with and without inclusion of the phase variables (employing phase analysis cut-off points determined from the ROC analysis noted in Table 4). When including entropy to the model, the area under the curve (AUC) for the detection of scar improved from 0.62 to 0.69 (*p* = 0.005) (Fig. 2).

**Table 5:** Multivariable analysis of the phase analysis parameters

Variable	OR	LCL	UCL	*p* value
Derivation cohort—clinical/imaging variables^a^ alone				
Clinical/imaging variables	1.326	1.132	1.552	0.0005
Derivation cohort—clinical/imaging variables^a^ + entropy				
Clinical/imaging variables	1.322	1.121	1.559	< 0.0001
Entropy (≥ 59%)	5.244	2.856	9.629	0.0009
Derivation cohort—clinical/cmaging variables^a^ + phase histogram bandwidth				
Clinical/imaging variables	1.370	1.162	1.615	< 0.0001
Phase histogram bandwidth (≥ 78°)	4.186	2.386	7.345	0.0002
Derivation cohort—clinical/imaging variables^a^ + phase standard deviation				
Clinical/imaging variables	1.331	1.133	1.563	0.0006
Phase standard deviation (≥ 26.7 °)	3.084	1.619	5.875	0.0005

*Abbreviations*: *LCL* lower confidence interval, *OR* odds ratio, *UCL* upper confidence interval

^a^See methodology section for discussion of chosen clinical/imaging variables to include in model

### Validation cohort

**Fig. 3:**
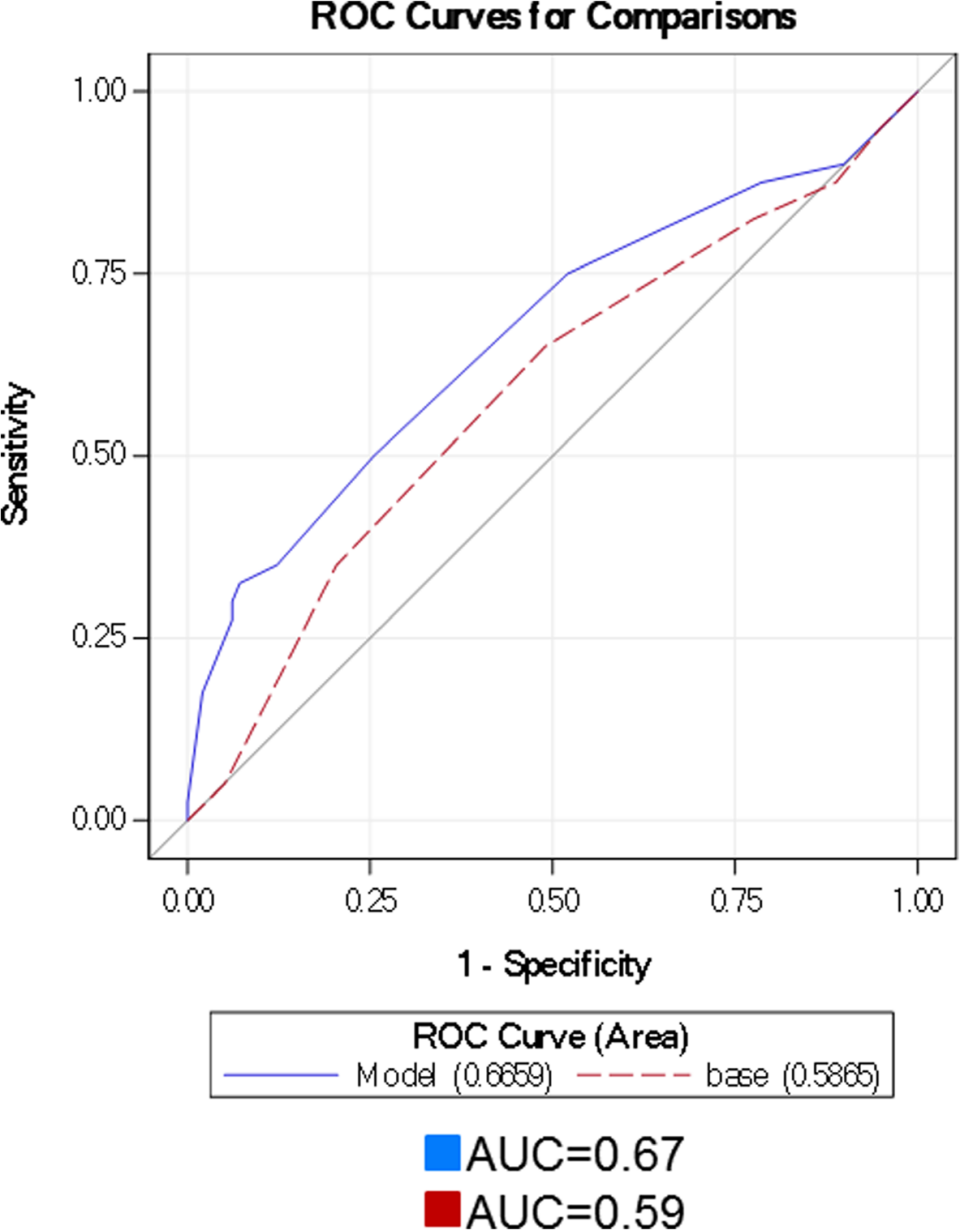
AUC difference estimate, 0.08 (0.002, 0.16), *p* = 0.04. *Abbreviations*: AUC area under the curve, NRI net reclassification improvement, ROC receiver operator curve

Forty patients (29%) in the validation cohort had scar, and 98 (71%) did not have scar as detected by SPECT MPI. The number of patients with scar as detected by SPECT MPI was not different between the two cohorts (31% vs. 29%, *p* = 0.68). Importantly, entropy (≥ 59%) remained independently associated with scar on multivariable analysis of the validation cohort with an OR of 5.96. Furthermore, addition of entropy to the clinical/imaging model improved the AUC from 0.59 to 0.67 (Fig. 3) with a total study population NRI of 10% (*p* = 0.04). For the 98 patients without scar, the addition of entropy (< 59) to the clinical/imaging model correctly reclassified 23 (24%) patients to a lower risk category, incorrectly classified 5 (5%) patients to a higher risk category, and did not change classification for the remaining 70 (71%) of patients resulting in a NRI of 18 (*p* = 0.04). For the forty patients with scar, 6 (15%) were correctly reclassified to a higher risk category, 9 (22.5%) were incorrectly reclassified to a lower risk category, and 25 (62.5%) were not reclassified with a resultant NRI of − 8%.

## Discussion

This study shows the potential of PA as an adjuvant tool for the accurate detection of myocardial perfusion defects in patients with known or suspected CAD. To our knowledge, the current investigation is the largest study that uses PA to assess myocardial scar utilizing resting SPECT MPI and is the first study to demonstrate its impact on patient reclassification.

When considering the prognostic and therapeutic implications of diagnosing a patient as having myocardial scar [5, 8], it is critical to avoid “false-positive” reports which may actually be attributable to artifacts. Phase analysis SPECT MPI is an automated, quantitative, repeatable, and reproducible means by which to assess LV dyssynchrony during SPECT MPI without the need for additional imaging time or radiation exposure. Until recently, the predominant areas of investigation utilizing PA have been in assessing its ability to predict responders to cardiac resynchronization therapy [23]. However, emerging literature has suggested it may be a potentially useful adjunct to SPECT MPI in the evaluation of patients with known or suspected CAD. In fact, previous studies have shown that PA can aid in the detection of ischemia by demonstrating worsening of dyssynchrony after stress testing in patients with CAD [22, 38–42]. Specifically, these investigations demonstrated that both thallium-201 as well as ^99m^Tc stress studies detected dyssynchrony in patients with myocardial perfusion defects as measured by PHB and PSD [38, 40]. Furthermore, the degree of ischemia (multivessel disease vs. non-multivessel disease) impacted the extent of dyssynchrony [39]. Finally, dyssynchrony parameters could be utilized to aid in differentiating between ischemic and non-ischemic cardiomyopathy [41]. The current investigation is the largest known study that uses PA to assess myocardial scar utilizing SPECT MPI and is the first study to demonstrate its impact on patient reclassification specifically when compared to expert analysis combined with automated SRS and IQ scores. Our study found that when added to clinical/imaging variables, the use of entropy has a significant impact on patient reclassification (Table 6).

**Table 6:** Patient reclassification when applying entropy

Patient group (*N*)	Higher risk (%)	Lower risk (%)	No change (%)	NRI (%)
Scar (40)	6 (15)	9 (22.5)	25 (62.5)	− 8
No scar (98)	5 (5.1)	23 (23.5)	70 (71.4)	18

There is a paucity of data on the potential benefit of PA for interpretation of rest myocardial perfusion defects. The findings of the current study of a greater degree of dyssynchrony in patients with scar are consistent with the limited data available of PA on resting perfusion imaging [38, 40, 42]. To have broader clinical applicability and to represent a better cross-section of the clinical population, the current investigation included patients with LBBB, paced rhythm [40, 42], prior coronary revascularization [42], atrial fibrillation, and cardiomyopathy.

The objective of the study was to assess the potential use of PA variables to improve SPECT MPI’s specificity. The current study highlights the potential role of PA, specifically entropy, for the correct interpretation of MPI studies in patients undergoing assessment for CAD. Entropy not only had a strong independent association with scar but also improved the AUC when compared to traditional/clinical imaging variables. As indicated in the statistics section, and by protocol design, PA variable thresholds were optimized to improve specificity and limit “false-positive” reporting of scar and not to improve sensitivity (already high with MPI). Other PA variables, like PHB and PSD, were also independently associated with scar in the derivation cohort, but this significance was not maintained in the validation cohort. One potential etiology for this finding would be the smaller patient population assessed in the validation cohort. Potential future investigations could address this limitation with the inclusion of a larger validation study population. Lastly, the current study found that entropy demonstrates a high rate of correctly reclassifying patients as having a lower probability of scar.

**Fig. 4:**
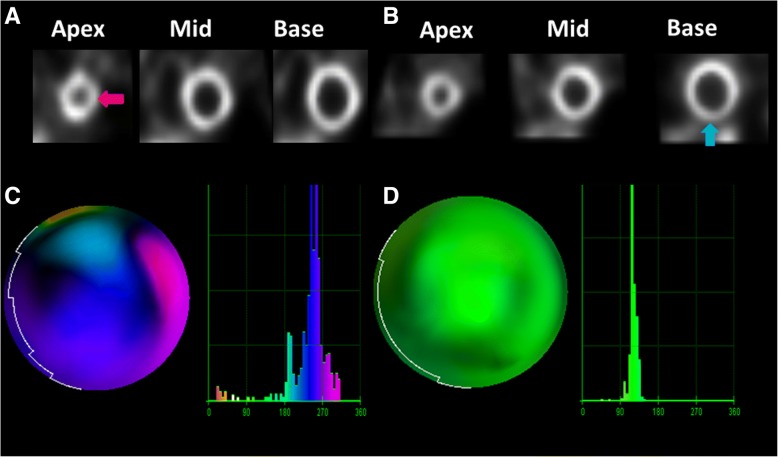
Comparative cases demonstrating use of phase analysis in instances of questionable resting defect (scar). A 74-year-old male with a history of hypertension, hyperglycemia and obstructive sleep apnea presented with 4 months of exertional dyspnea and palpitations was referred for radionuclide stress testing. Short-axis rest and stress images displaced from apex to base (**a**) demonstrated a possible resting perfusion defect in the lateral portion of the apex (red arrow). **b** A 76-year-old obese, hypertensive male presented with complaints of 3 months of exertional dyspnea underwent radionuclide stress testing which revealed a possible resting defect in the basal inferior segment (blue arrow). **c** Resting polar map and histogram of patient from case (**a**) demonstrating significant dyssynchrony, with an entropy 72%. Compare these findings with the polar map and histogram (**d**) of the second patient (**b**) which demonstrates synchrony with entropy 45%

From the clinical application perspective, we envision the potential use of dyssynchrony thresholds derived from this study as a means by which the interpreting nuclear physician can assess whether the presumed resting perfusion defect truly exists or whether it might simply be artifactual (Fig. 4). Specifically, if there is doubt in regards to the potential veracity of a resting defect on MPI, a normal PA entropy (< 59%) could serve as a means by which to further clarify that scar is not present. Furthermore, the impact of PA entropy in determining myocardial scar would likely be even greater in laboratories with a single reader who does not have access to an automated IQ program and who is without access to all relevant clinical data. Ultimately, a combination of the high specificity of PA with the high sensitivity of MPI could render the use of SPECT-MPI as an ideal diagnostic tool for the evaluation of patients with CAD. Finally, specific subpopulations, particularly patients classified as having heart failure with reduced ejection fraction, where the detection of myocardial scar is of critical prognostic and therapeutic importance would benefit from enhanced accuracy in the detection of scar.

### Limitations

This study was conducted at a tertiary referral center, resulting in potential referral and selection bias. However, the baseline demographics of the study population (69% male, mean age 67 years) are consistent with other study populations undergoing nuclear cardiac stress testing [43]. The current PA assessment was performed utilizing a specific software (QGS 3.0; Cedars-Sinai Medical Center, Los Angeles CA) whereas other investigations have used different software packages (such as the SyncTool™ and Emory Cardiac Toolbox). Despite these differences, these studies [38, 40, 42] have noted similar results to the current study. That being said, the defined cut-off points for dyssynchrony that were determined in the current study are possibly unique to this software, and caution should be exercised in the extrapolation of specifics of this measurement to other packages. Some software packages also do not have all PA measurements reported in the current study, and method of acquisition of PA parameters may differ between vendors, limiting the generalizability of these results. Furthermore, we used image acquisition and processing protocols specific to our DSPECT MPI laboratory that may be different from other centers. However, as previously mentioned, prior studies have demonstrated that PA is repeatable [18] and reproducible [17], with results that are independent of the imaging system [20], reconstruction algorithm [21], or tracer activity utilized [22].

In the current study, resting perfusion defect was labeled scar but some of these patients may have actually had viable myocardium. Nitrate administration may have helped further discriminate between these two populations. However, both populations reflect patients with abnormal myocardium and potential CAD, and thereby discerning between these patients and those with completely normal myocardial perfusion still has diagnostic, prognostic, and therapeutic implications.

In this study, we did not include an independent measurement of scar, but rather depended on clinical assessment, as done in routine practice. One potential consideration could be the utilization of gadolinium delayed-enhancement cardiac magnetic resonance imaging (cMRI) to assess scar in patients also undergoing PA SPECT MPI. This would be of importance in determining the potential role of PA, specifically entropy, when discordance arises between interpreters who are convinced that scar is present and the PA data which suggests otherwise.

Finally, comparative assessment with segmental wall motion and thickening was not performed and would potentially provide further insights when compared to phase analysis parameters.

## Conclusion

The use of PA entropy significantly improved the specificity of SPECT MPI using a solid-state system in a center with dual physician SPECT MPI interpreters, a quantitative QI program, and ready access to relevant clinical data. It would likely have even greater impact in a center with less readily available resources and thereby serve as a useful adjunctive tool to the nuclear cardiologist.

## Abbreviations

^99m^Tc: Technetium-99m
AUC: Area under the curve
CAD: Coronary artery disease
cMRI: Cardiac magnetic resonance imaging
LBBB: Left bundle branch block
LV: Left ventricular
MELM: Maximum-likelihood expectation-maximization
MPI: Myocardial perfusion imaging
NRI: Net reclassification index
OMC: Onset of myocardial contraction
PA: Phase analysis
PHB: Phase histogram bandwidth
PSD: Phase standard deviation
QGS: Quantitative gated SPECT
QPS: Quantitative perfusion SPECT
ROC: Receiver-operator characteristic
SPECT: Single-photon emission computed tomography
SQ: Scar quantification
SRS: Summed rest score
